# Temporal characterization of acute pain and toxicity kinetics during radiation therapy for head and neck cancer. A retrospective study

**DOI:** 10.1016/j.oor.2023.100092

**Published:** 2023-08-23

**Authors:** Vivian Salama, Sara Youssef, Tianlin Xu, Kareem A. Wahid, Jaime Chen, Jillian Rigert, Anna Lee, Katherine A. Hutcheson, Brandon Gunn, Jack Phan, Adam S. Garden, Steven J. Frank, William Morrison, Jay P. Reddy, Michael T. Spiotto, Mohamed A. Naser, Cem Dede, Renjie He, Abdallah S.R. Mohamed, Lisanne V. van Dijk, Ruitao Lin, Carlos J. Roldan, David I. Rosenthal, Clifton D. Fuller, Amy C. Moreno, MD Anderson Head and Neck Cancer Symptom Working Group

**Affiliations:** a Department of Radiation Oncology, The University of Texas, MD Anderson Cancer Center, Houston, TX, USA; b Department of Biostatistics, The University of Texas, MD Anderson Cancer Center, Houston, TX, USA; c Department of Head and Neck Surgery, The University of Texas, MD Anderson Cancer Center, Houston, TX, USA; d Department of Radiation Oncology, Medical Center Groningen, University of Groningen, Groningen, NL, USA; e Department of Pain Medicine, Division of Anesthesiology, Critical Care Medicine, and Pain Medicine, The University of Texas, MD Anderson Cancer Center, Houston, TX, USA

**Keywords:** Head and neck cancer, Oral Cavity and oropharyngeal cancer, Acute pain, Radiation Therapy, Oral mucositis, Dermatitis

## Abstract

**Objectives:**

Pain during Radiation Therapy (RT) for oral cavity/oropharyngeal cancer (OC/OPC) is a clinical challenge due to its multifactorial etiology and variable management. The objective of this study was to define complex pain profiles through temporal characterization of pain descriptors, physiologic state, and RT-induced toxicities for pain trajectories understanding.

**Materials and methods:**

Using an electronic health record registry, 351 OC/OPC patients treated with RT from 2013 to 2021 were included. Weekly numeric scale pain scores, pain descriptors, vital signs, physician-reported toxicities, and analgesics were analyzed using linear mixed effect models and Spearman’s correlation. Area under the pain curve (AUC*pain*) was calculated to measure pain burden over time.

**Results:**

Median pain scores increased from 0 during the weekly visit (WSV)-1 to 5 during WSV-7. By WSV-7, 60% and 74% of patients reported mouth and throat pain, respectively, with a median pain score of 5. Soreness and burning pain peaked during WSV-6/7 (51%). Median AUC*pain* was 16% (IQR (9.3–23)), and AUC*pain* significantly varied based on gender, tumor site, surgery, drug use history, and pre-RT pain. A temporal increase in mucositis and dermatitis, declining mean bodyweight (−7.1%; *P* < 0.001) and mean arterial pressure (MAP) 6.8 mmHg; *P* < 0.001 were detected. Pulse rate was positively associated while weight and MAP were negatively associated with pain over time (*P* < 0.001).

**Conclusion:**

This study provides insight on in-depth characterization and associations between dynamic pain, physiologic, and toxicity kinetics. Our findings support further needs of optimized pain control through temporal data-driven clinical decision support systems for acute pain management.

## 1. Introduction

Acute pain is a commonly debilitating symptom experienced by patients with oral cavity or oropharyngeal cancer (OC/OPC) even before undergoing definitive or post-operative radiation therapy or concurrent chemoradiation (RT/CRT). Effective management of pain during RT/CRT is challenging due to a heterogeneous patient population, its multifactorial etiology, and lower than anticipated responsiveness to opioids [1,2]. In general, over 90% of head and neck cancer (HNC) patients report acute pain with 50–80% requiring opioid therapy [3–5]. While the World Health Organization (WHO) offers “analgesic ladder” guidelines for stepwise medication intensification [6–9], it is neither patient nor pain syndrome-specific, resulting in a relative paucity of analgesia effectiveness with approximately one-third of cancer patients still presenting to the emergency department with uncontrolled pain as a chief complaint [10–13]. Whereas the presence of pre-treatment pain, an independent predictor of survival, organ failure, the inability to use the oral route, polypharmacy, anxiety and psychiatric disorders, addiction, allergies, poor tolerance to medications, in addition to the lack of insurance coverage are additional challenges to overcome to provide adequate pain control during RT/CRT [14]. Published studies suggest pathophysiologic mechanisms of pain with varying clinical manifestations [15,16]. Pain characterization as inflammatory, nociceptive-somatic, nociceptive-visceral, and neuropathic have been reported using different symptom attributes such as “burning sensation”, to describe inflammatory pain, or “burning, numbness, shooting, or stabbing” for neuropathic pain [17–20]. However, in cancer patients including those undergoing RT/CRT, pain is considered mixed, with overlapping nature.

The ability to adequately characterize and differentiate mechanistic properties of acute pain experienced by OC/OPC patients during RT/CRT may facilitate symptoms management, thus alleviating morbidity and reducing the cost of care. While HNC pain-related studies exist in the literature [1,15,21,22], they are often limited by their broad scope (i.e., generalized multi-symptom burden analysis) or focused on chronic pain and/or opioid dependency at a specified time *after* oncologic therapy. These studies also lack the inclusion of pain descriptors and physiologic kinetics (i.e., vital signs) which may elucidate underlying mechanisms of acute pain.

To address this unmet need, the objective of this study was to provide an in-depth analysis on dynamic, acute pain profiles experienced by a modern, prospective OC/OPC cohort treated with curative RT/CRT. Temporal characterization of vital signs, provider-based treatment-related toxicity assessments [23], and analgesic prescription patterns during RT/CRT are also reported.

## 2. Methods

### 2.1. Study procedures and population

This study was approved by the Institutional Review Board of The University of Texas MD Anderson Cancer Center (UT-MDACC).

A radiation oncology electronic data capture system (Brocade) developed at UT-MDACC, which is EHR-interoperable and allows for prospective clinical data collection during RT and populates to the EHR (i.e., Epic), was used for cohort identification [24]. This secure database was queried for patients diagnosed with squamous cell carcinoma (SCC) of the OC/OPC, or metastatic to the neck with unknown primary, who were treated with curative-intent RT/CRT from 2013 to 2021. RT prescriptions including 60–70 Gy in about 30–33 fractions were included, and modalities included intensity-modulated radiotherapy (IMRT), volumetric modulated arc therapy (VMAT), and proton therapy. OC cancer patients had surgery with postoperative RT or CRT while OPC patients were treated primarily with definitive RT/CRT. All patients included must had documented toxicity data and reportable pain score data from at least 3 weekly see visits (WSVs).

### 2.2. Cohort characteristics and study measures

Patient demographics, exposure history (i.e., smoking, alcohol, illicit drugs), clinical stage per AJCC (American Joint Committee on Cancer) 7th edition or 8th edition for recent patients [25,26], RT details, and WSV subjective (i.e., reported by patient) and objective (i.e., reported by provider) data were extracted from Brocade in addition to our EHR for any additional collection of baseline pain scores (i.e., prior to RT/CRT) and WSV-based data on analgesic prescriptions and nursing assessments of pain.

Temporal pain measures extracted included patient-reported pain scores using a numeric rating scale (NRS, scores of 0–10) and qualitative parameters such as pain location (i.e., mouth, throat, skin), description (i.e., aching, sore, burning, sharp), onset and progression. Pain trajectories were reported as occurring at baseline or during WSV# (i.e., WSV6 signifies the 6th WSV). Of note, some results focus on WSV6 as “end of treatment” as it captures all OC/OPC cases treated to fraction 30 (i.e., mainly patients receiving >31 fractions underwent a WSV7 assessment).

WSV-based objective toxicity measures collected include physician-graded mucositis and dermatitis reported using the National Cancer Institute Common Terminology Criteria for Adverse Events (version 5.0; CTCAE) [23]. Provider-based overall treatment toxicity assessments (i. e., absent, mild, moderate, or severe) were also extracted.

Weekly physiologic parameters extracted from the EHRs included weight (WT: kg), blood pressure (BP, mmHg), and heart rate (HR, beats/min). The mean arterial pressure (MAP: mmHg) was calculated using the following formula: MAP = (systolic pressure + 2*diastolic pressure)/3 [27]. Percent weight change was calculated using the following equation: 100*[(current weight-baseline weight)/baseline weight].

Prescribed analgesics data and the cumulative analgesics used by patients was categorized into the following groups: topical analgesics (lidocaine/Xyloxylin); systemic analgesics including non-opioids (i.e., non-steroidal anti-inflammatory drugs); weak, commonly referred as codeine-based opioids such as codeine, hydrocodone, and dihydrocodeine, and tramadol [9,28]; strong opioids including morphine and semisynthetic/synthetic agents such hydromorphone, fentanyl and methadone [28]; and gabapentin. A categorization of weak versus strong opioids has been used in other studies [8,29,30].

### 2.3. Statistical analysis

Temporal characterization of pain scores, weight, and vitals were assessed using one-way ANOVA tests. Trends in temporal pain scores and vital signs were assessed using a linear mixed-effect model (LMM) with random intercepts, accounting for associations between repeated measurements in the same subject. The study time period (i.e., during RT/CRT) was treated as a fixed effect and the subject as a random effect. Spearman’s rank correlation was used to calculate the correlation between changes in pain and physiologic measures. Differences in treatment toxicities and opioid prescribing patterns were analyzed using the Chi-square test. AUC*pain* scores were calculated using previously described methods [31]. Briefly, the area under the curve of temporal pain scores was calculated and divided by the total area (maximum pain score × number of followed weeks). AUC pain metrics were then compared using the Wilcoxon rank-sum test for binary variables and Kruskal-Walli’s test for multilevel variables based on gender, age (≥ 60 years old or <60 years old), primary tumor site, exposure history, receipt of chemotherapy or surgery, and pre-RT pain score. The Wilcoxon rank-sum test was used for binary variables of pain scores between the analgesics’ groups.

All statistical tests were 2-sided with statistical significance indicated at *P* < 0.05. Analyses were performed using graph pad prism (version 8), JMP PRO 15, and R statistical software (version 4.0.3).

## 3. Results

### 3.1. Patient characteristics

Patient characteristics are outlined in Table 1. We identified 351 OC/OPC patients [OC (n = 120, 34%), OPC (n = 228, 65%) and unknown primary (n = 3, 1%)] treated with RT/CRT. The mean age at diagnosis was 58.5 years (range, 21–83 years, SD 10.8), and most were White (n = 302, 86%) and male (n = 261, 74%). Approximately two-thirds had HPV-positive cancer (59%) and a clinical T stage of T1-T2 (62%), whereas half had N2 adenopathy (n = 170, 49%). Surgery was used in 58% of the cohort (97.5% of OC (n = 117), and 13.6% of OPC), 14% received induction therapy, and 56% were treated with CRT.

### 3.2. Characterization of acute pain

**Fig. 1.**
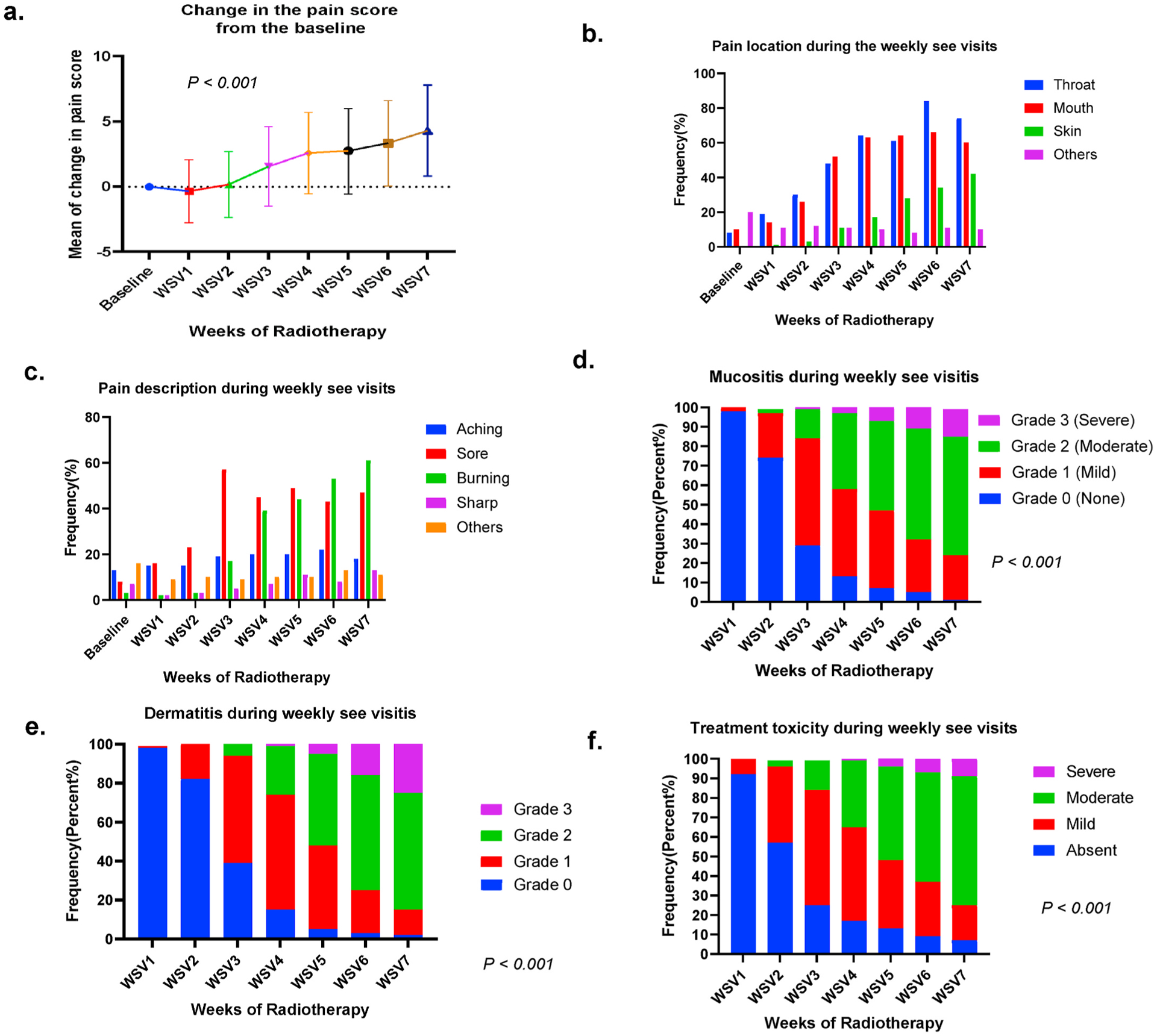
Acute pain profile and acute toxicities: (a) Overall change of patient reported pain score (delta pain) showed significant increase in the mean of pain score during the weeks of RT (WCVs) (*P* < *0.001*). (b) Overall pain location reported in the weekly see visits. (c) Overall pain description reported in the weekly see visits. (d) Physician reported mucositis grades showed significant increase over the weeks of RT (*P* < *0.001*). (e) Physician reported dermatitis grades showed significant increase over the weeks of RT (*P* < *0.001*). (f) Physician reported overall treatment toxicity plan showed significant increase over the weeks of RT (*P* < *0.001*).

Longitudinal pain profiles are summarized in Table 2. A total of 341 (97.2%), 351 (99.7%), 303 (87%) and 159 (45.3%) patients reported any pain score at baseline, WSV1, WSV6, and WSV7, respectively. There was a temporal progressive increase in mean (median) pain scores from a baseline of 1.4 (0), to 4.7 (5), on WSV7. The overall change in pain intensity from WSV1 to WSV6 was significant (*P* < *0.001*, Fig. 1a). By WSV6, 66% and 84% of the cohort reported mouth and throat pain, respectively, with a median pain score of 5 (range, 1–10). Skin-related pain started at WSV1 (n = 5, 1%) with a steady increase to 34% by WSV6 (Fig. 1b).

Pain descriptors during RT reflected almost a consistent scoring of “aching” sensation throughout the WSVs (13%–22%), while “soreness” and “burning” demonstrated the greatest temporal changes (Fig. 1c). “Sore” pain was reported in 8% (n = 28) of patients’ pain descriptors at baseline, peaked at 57% (WSV3), and decreased to 43% by WSV6. “Burning” pain was less common at baseline (3%) and the first 2 WSVs but escalated to 17% by WSV3 with a peak at WSV7 (61%). The pain frequency was predominantly “intermittent” during RT WSV1 (10%) with a gradual shift towards being “constant” by end of WSV7 (45%). This coincided with a gradual worsening of pain from WSV1 (11%) to WSV7 (40%) with minimal gradual/rapid improvement despite delivered analgesics.

**Fig. 2.**
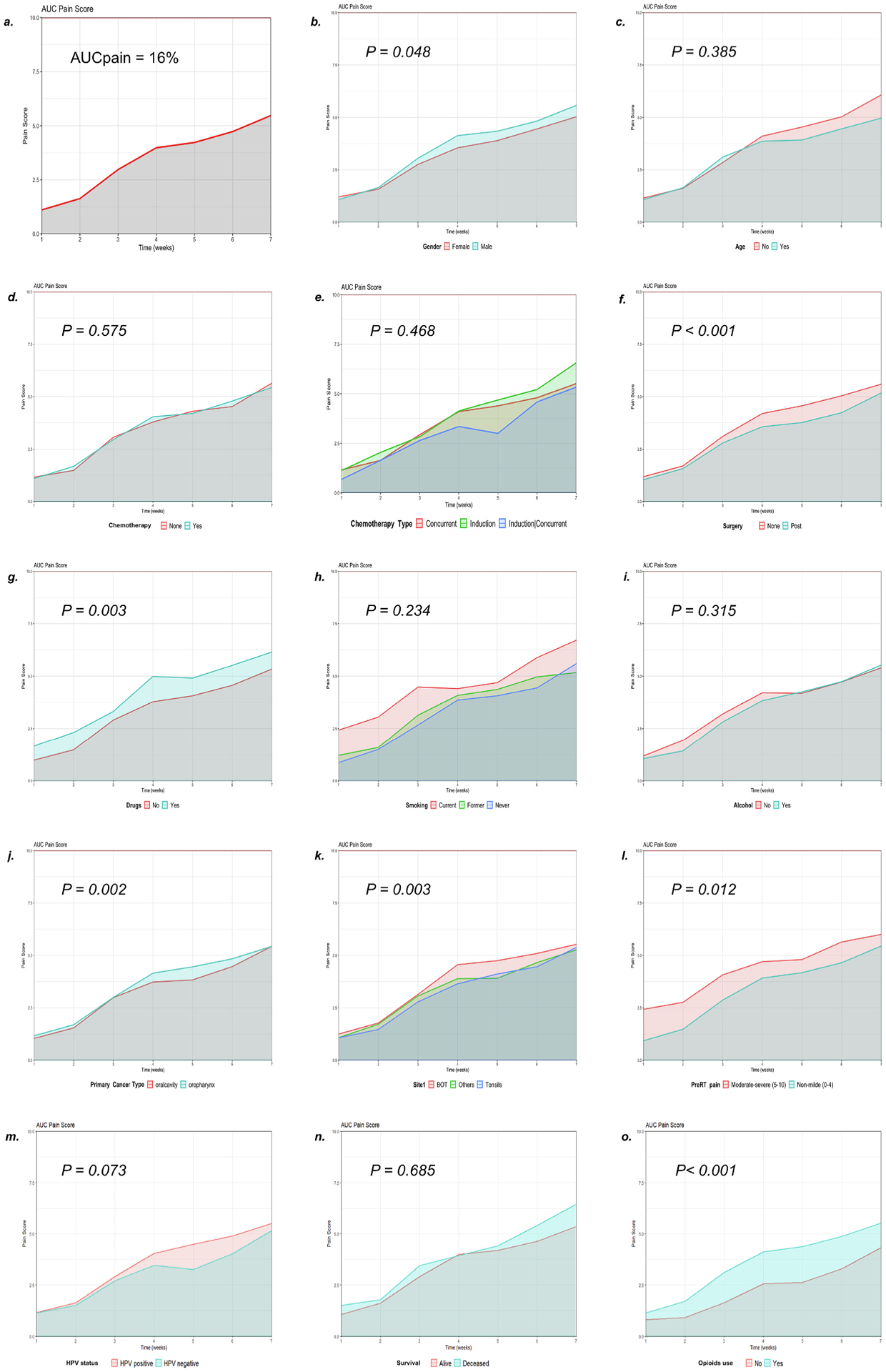
AUC*pain* scores **a.** Overall pain AUC curve (16%). **b.** AUC*pain* scores based on gender, male group had significant higher AUC pain curve than female (***P*** =***0.048***). **c.** AUC*pain* scores based on age of 60 years, no significant difference between < 60 (No) and >=60 (Yes) years old (*P* = *0.385*). **d.** AUC*pain* scores of systemic medication (chemotherapy) use showed no significant difference (*p*=*0.575*). **e.** AUC*pain* scores based on chemotherapy type showed no significant difference (*P* =*0.468*). **f.** AUC*pain* scores based on pre-RT surgery, patients had RT post-surgery (Post) had significant lower pain curve than patients did not have surgery (None) (***P* < *0.001***). **g.** AUC*pain* scores based on drug abuse, patients had history of drug abuse had significant higher pain curve than patients did not (No) (***P*** = ***0.003***). **h.** AUC*pain* scores based on smoking status showed no significant difference (*p* = *0.234*). **i.** AUC*pain* scores based on alcohol status showed no significant difference (*P* = *0.315*). **j.** AUC*pain* scores based on primary cancer type, oropharynx group had significant higher curve than oral cavity (***P*** = ***0.002***). **k.** AUC*pain* scores based on primary cancer site, Base of tongue group had significant higher curve than tonsils and others (***P*** = ***0.003***). l. AUC*pain* scores based on pre-RT pain scores, group had non-mild pain score (0–4) had lower curve than group with moderate-severe (5–10) pain (***P*** = ***0.012***). **m**. AUC*pain* scores based on HPV status showed no significant difference between HPV positive and HPV negative (*p* = *0.073*). **n.** AUC*pain* scores based on survival status showed no significant difference between Alive and Deceased patients (*P* = *0.685*). **o.** AUC*pain* scores based on opioids use, patients used opioids (Yes) had significant higher pain curve than patients did not use opioids (No) (***P* < *0.001***).

We condensed temporal information related to pain trajectories into a single metric, AUC*pain* scores, which were calculated for the entire cohort (Median 16.0% (IQR (9.3–23.0)) and for specific subgroups. Significant difference in AUC*pain* scores in subgroups based on gender (median male 16.5% (IQR 9.5–24.5), female 13.8% (IQR 8.5–19.9), *P* =*0.048*), primary cancer type (median oral cavity 14.5% (IQR 7.5–20.0), oropharynx 17.5% (10.4–25.0), *P* = *0.002*), primary tumor site (BOT 19.8% (IQR 12.5–25.6), tonsils 14.0% (IQR 8.5–21.1), others 14% (IQR 9.0–21.6), *P* = *0.003*), surgery (none 17.5% (IQR 11.0–25.0), post 12.5% (6.0–20.0), p < 0.001), drug abuse (No 14.5% (IQR 9–22), Yes 20.8% (IQR 12.4–26.3), *P* = *0.003* and pre-RT pain score (non-mild (score 0–4) 20.3% (12.1–30.0), moderate-severe (score 5–10) 15.5% (9.0–22.3), *P* = *0.012*) while no significant difference was detected in subgroups based on chemotherapy received (*P* = *0.468*), age (*P* =*0.385*), smoking (*P* = *0.234*), alcohol history (*P* = *0.315*), HPV status (negative 13.25% (IQR 7.75-19-75), positive 16.75% (IQR 10–24.88), *P* = *0.073*) or the survival status (Alive 16% (IQR 9–23), Deceased 15% (IQR 10.50–24.25), *P* = *0.685*) (Fig. 2a–n).

### 3.3. Provider-based assessments

Weekly mucositis and dermatitis scores showed a significant increase in severity over time (*P* < *0.001*). There was a sharp decline in grade 0 mucositis from WSV2 (74%) to WSV3 (29%) corresponding with increases in grades 1, 2, and 3 mucositis with progressive severity peaking at WSV7 where rates of grade 0, 1, 2, and 3 mucositis were 1%, 23%, 61%, 14%, respectively (Fig. 1d.). Similar trajectories in dermatitis grading were noted with grade 2+ dermatitis increasing from 0% during WSV1 to 26%, 75%, and 85% during WSVs 4, 6, and 7, respectively (*P* < *0.001*; Fig. 1e.). From a clinician’s perspective, overall treatment toxicity assessment during WSV7 was reportedly “moderate” in 66% of the cohort while “severe” in only 9% of the cohort (Fig. 1f.).

### 3.4. Physiologic kinetics

**Fig. 3.**
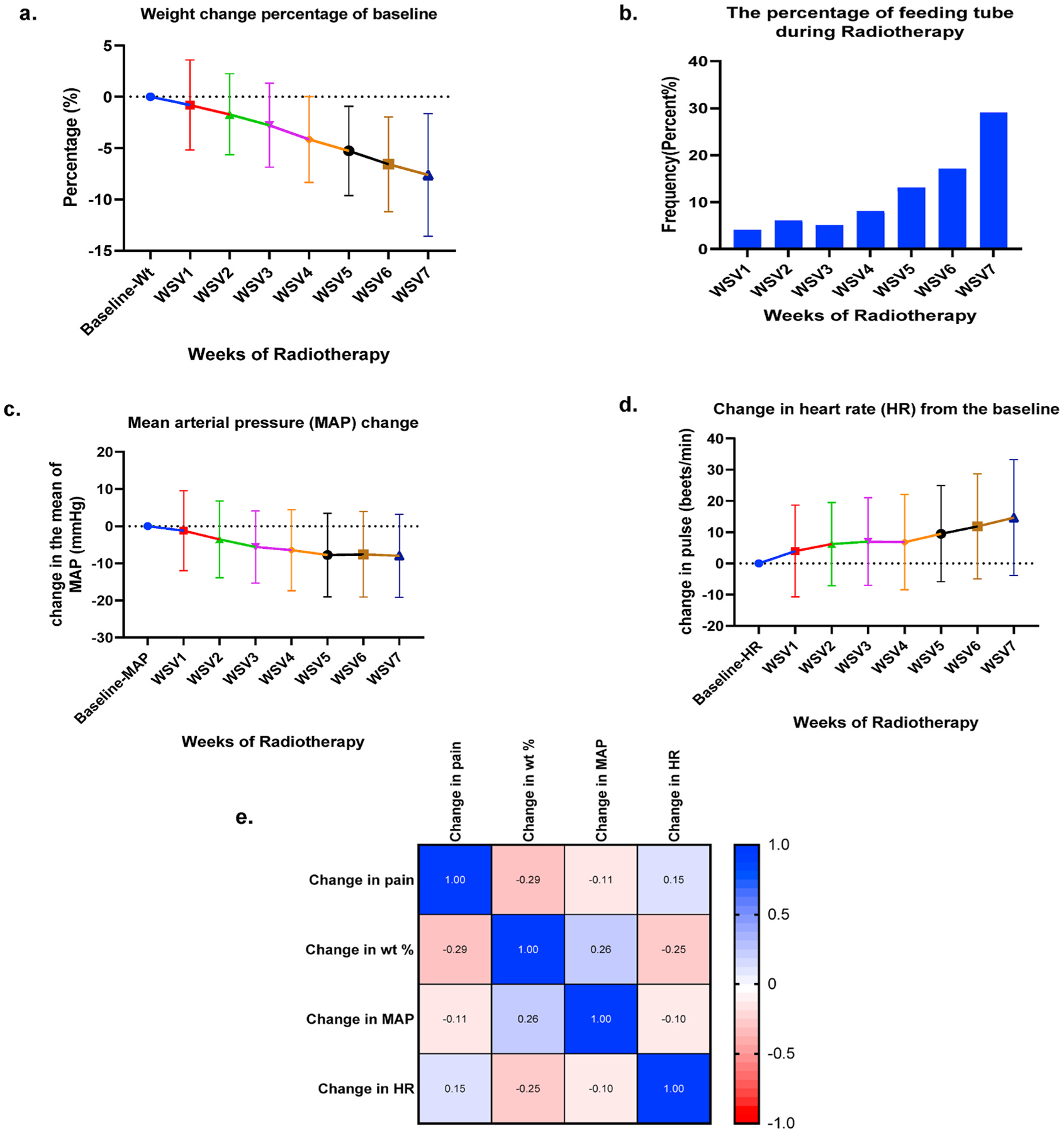
Physiologic changes: (a) temporal overall change in the mean of body weight change during the weeks of RT. (b) Frequency of feeding tube used through the weekly see visits of RT. (c) Temporal change in the mean of mean arterial pressure (MAP) change during the weekly see weekly see visits. (d) Temporal change in heart rate (HR) change during the weekly see visits. (e) Spearman r correlation matrix showing correlation between the change in pain and the change in vital signs.

A significant percent body weight decline of 7.1% for the entire cohort was seen by the end of RT (95% CI, 9.96–8.17; *P* < *0.001*) (Fig. 3a.). An increase in the rate of feeding tube insertions during RT was detected, with 4% of patients having a feeding tube at WSV1 compared to 13% ((n = 32, 13%) of patients received CRT and (n = 12, 11.7%) of patients received RT alone) and 29% ((n = 32, 30%) of patients received CRT and (n = 3, 16%) of patients received RT alone) during weeks 5 and 7 (*P* < *0.001*; Fig. 3b.). A MAP drop of 6.8 mmHg and a corresponding increase in mean HR by 10.7 beats/min (95% CI, 7.56–13.82; *P* < *0.001*) was observed from WSV1 to 7 (*P* < *0.001*, Fig. 3c–d.).

Using a Spearman r correlation (Fig. 2e), a positive association with time was noted for pain score changes (95% CI, 0.7–0.8; *P* < 0.001) and HR change (95% CI, 1.20–1.82); *P* < *0.001*) while a negative correlation was seen with weight (95% CI, −1.29 to −1.10; *P* < *0.001*) and MAP change (95% CI, −6.29 to −4.90; *P* < *0.001*). Additionally, a positive correlation between pain and HR (r = 0.15, 95% CI, 0.11–0.19; *P* < *0.001*) as well as between weight and MAP (r = 0.26, 95% CI, 0.22–0.30; *P* < *0.001*) were observed. A negative correlation was detected between pain and weight (r = −0.29, 95% CI, −0.32 to −0.25; *P* < *0.001*), pain and MAP (r = −0.12, 95% CI, −0.15 to −0.07; *P* < *0.001*), weight and HR (r = −0.25, 95% CI, −0.29 to −0.21; *P* < *0.001*) and MAP and HR (r = −0.10, 95% CI, −0.14 to −0.06; *P* < *0.001*) (Fig. 3e.).

### 3.5. Analgesics/opioid prescribing patterns

**Fig. 4.**
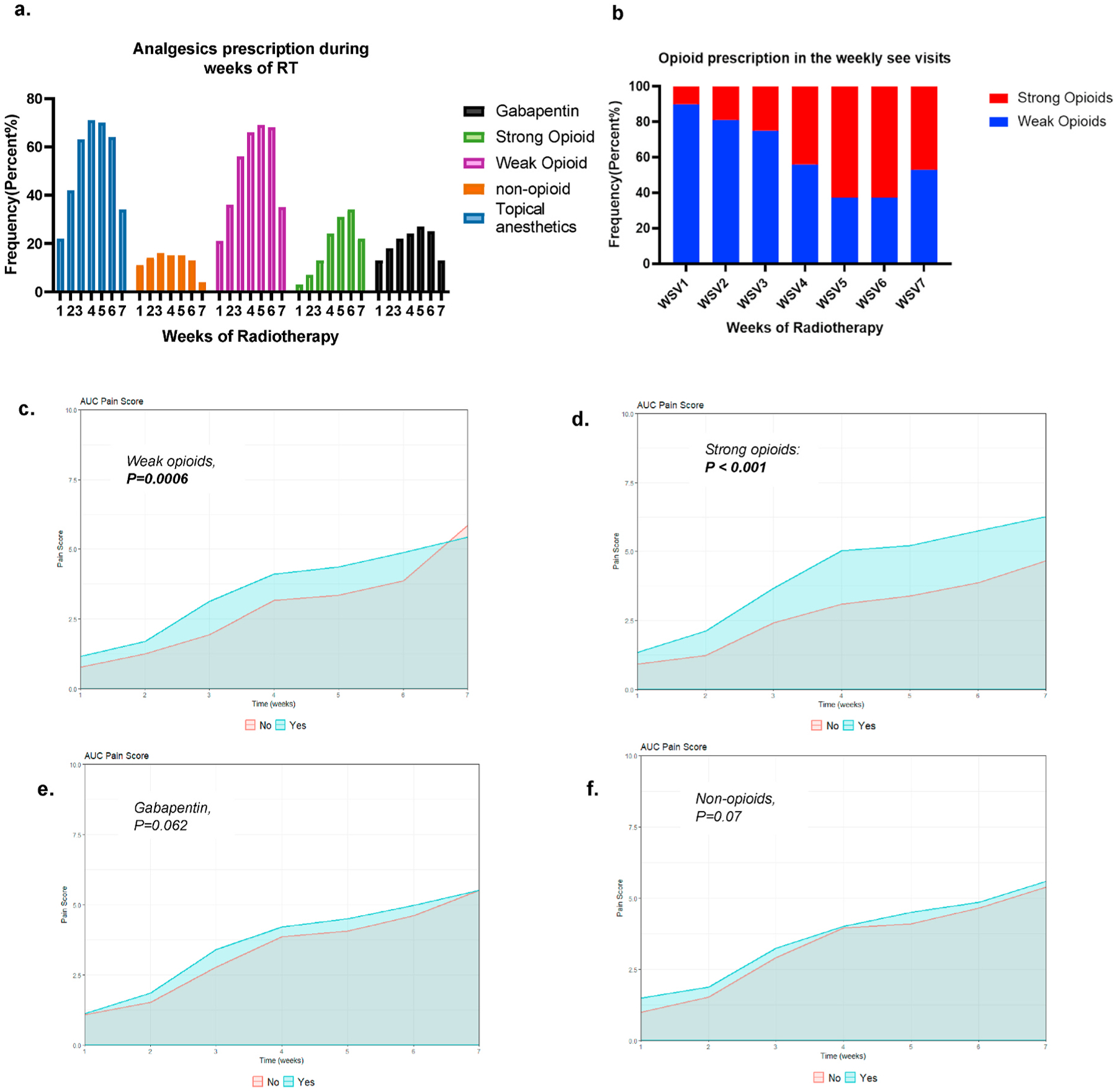
(a) Temporal cumulative frequency of analgesics used by patients during the weekly see visits (WSVs) of radiotherapy. (b) The frequency of weak opioids versus strong opioids prescribed during the WSVs shows significant difference in opioids prescription (*P* < *0.001*). (c) AUC*pain* scores in subgroups based on weak opioids use (*P* = *0.0006*). (d) AUCpain scores in subgroups based on strong opioids use (P < 0.001). (e) AUCpain scores in subgroups based on gabapentin use (*P* =*0.062*). (f) AUCpain scores in subgroups based on non-opioids use (*P* = *0.07*).

A temporal increase in the cumulative frequency of analgesics prescribed was detected throughout the weeks of RT (Fig. 4a). Topical analgesics and low dose or weak opioids were the most used agents, with peak use during WSV5 (70%) for topical analgesics and WSV6 (68%) for opioids. Higher dose or strong opioid use started during WSV1 at 3% and reached a peak of 34% during WSV6. Gabapentin use during WSV1 and WSV5 were 13% and 27%, respectively. A fivefold increase in strong opioid prescribing was noted over time from WSV1 (10%) to the end of RT (55%), concurrent with a temporal decrease in weak opioid (90% in WSV1 to 53% in WSV7; *P* < *0.001*). See Fig. 4b for details. Median patient reported pain levels among low dose or weak opioids users was 2 in WSV1, increased up to 4 by WSV4/5 reaching to 5 by WSV6/7 compared to non-users with median pain scores 0, 3, and 4 by WSV1, WSV4 and WSV6, respectively. Median pain levels among high dose or strong opioids users were 3 in WSV1 with sharp increase to 6 and 7 by WSV6 and WSV7, respectively, compared to non-users who reported median pain scores 0, 3, and 4 during WSV1, WSV5 and WSV6/7, respectively. There was no significant difference in median pain levels in gabapentin or non-opioid analgesics users compared to non-users (Table 3.). Analysis of pain trajectory among patients receiving opioids during treatment demonstrated significant higher AUC*pain* scores compared with patients who did not (Yes 17%, (10.5–23.5), No 7%, (IQR 4.0–13.0), *P* < *0.001*) (Fig. 2o.). Significant difference in AUC*pain* scores in subsets population based on weak or low dose opioids use during RT (Yes 16.75%, (IQR 10.38–23.62), No 9.5%, (IQR 4.75–18.25), *P* = *0.0006*), and high dose or strong opioids (Yes 20.5%, (IQR 15.00–28.38), No 11.5%, (IQR 6.50–19.00), *P* < *0.001*). However, no significant difference in AUC*pain* scores was detected in subgroups who used gabapentin or non-opioids, (P = 0.062) and (P = 0.07) respectively (Fig. 4c–f.) (Table 3).

## 4. Discussion

Acute pain is defined as a response of the nervous system to the presence of a harmful stimuli not extended beyond the healing process, typically with a duration of 3 months or less [32]. In this comprehensive analysis we aimed to review the patients’ characterization of acute pain, associated physiologic changes, and provider-based assessment and analgesic approaches during radiation therapy of head and neck cancer. At our institution, most radiation oncology providers implement a tiered approach for pain management that often includes gabapentin, non-opioids systemic and topical analgesics as the foundation (i.e., started during WSV1). As pain intensifies, low dose or weak, then high dose or strong opioid analgesics are frequently used. However, the high complexity of our patient population mandates an interdisciplinary management including both, non-interventional disciplines (Medical Oncology and Supportive care), as well as interventional (Surgical Oncology and Interventional Pain Management) especially in high-risk patients. Despite these strategic interventions and the escalation in opioids usage, this OC/OPC cohort showed a direct correlation between the increase in cumulative pain scores and the progression of RT-induced oral mucositis and dermatitis. Similarly, a correlation between pain severity and declining physiologic status was observed. Our findings are in alignment with rates of acute toxicities seen in recent randomized HNC clinical trials whereby grade 3–4 oral mucositis affected 42–46% of the cohort and 15% reported grade 3–4 acute pain [33]. Limitations to toxicity reporting in such trials included lack of temporal information or insight on how acute pain was managed.

Our group has previously reported on progressive symptom burden in a prospective longitudinal HNC cohort study using a validated patient-reported outcome (PRO) tool called the MD Anderson Symptom Inventory–Head and Neck Module (MDASI-HN) [1]. The current study builds upon those results by providing an in-depth analysis of acute pain, including novel reporting of AUC*pain* scores which have the advantage of consolidating time-series data into a single quantitative metric. Using calculations reported by van Dijk et al. [31], we found a significantly higher AUC*pain* burden in male patients and those who had a prior history of drug use, pre-therapy reported pain, no surgery (i.e., definitive RT/CRT), or a primary tumor site in the tongue base. Sex-based studies suggest a higher sensitivity to pain in females than males with differences in analgesic response being mixed or minimal between both sexes [34,35]. However, there are concerns that published studies are male-biased and not confirmed in female populations [36]. Interestingly with only 26% of our cohort being female (n = 90) (as 75% HNC patients are men), we found a statistically significant difference in pain trajectories, favoring increased pain sensitivity and scoring among men, not women. This may be related to sample size and distribution, but it may also reflect different acute pain mechanisms triggered in females [37] that are more readily identifiable when analyzing temporal pain data over central tendencies of pain scores. Similarly, worse AUC*pain* metrics in patients with pre-RT pain and/or substance abuse corroborate existing studies and reflect a higher-risk patient population [15,21,38]. Observed differences in AUC*pain* based on receipt of surgery may be dose-dependent (i.e., definitive non-surgical cases receive higher doses of radiation [15], versus nerve injuries acquired during surgery. Overall, the single AUC*pain* metric is a unique measure of longitudinal symptom burden that can potentially optimize future prediction models of treatment-related toxicities.

In our study, descriptions of pain as a “sore” or “burning” sensation escalated over time which may elucidate the known subjective intensity and characterization of some mechanisms behind the grading of mucositis and dermatitis. Several models of oral mucositis exist with radiation-induced activation of transcription factors such as nuclear factor kappa B and the release of several pro-inflammatory cytokines (i. e., interleukin (IL)-1β) playing critical roles in the ulceration phase [39, 40]. While “sore” and “aching” descriptors may indicate a component of inflammatory nociceptive pain, a “burning” characteristic is often reported with the pathobiology of neuropathic pain (a landmark of pain centralization, as a result of prolonged high intensity, or poorly controlled nociceptive stimuli) and/or underdiagnosed and mismanaged pain in HNC patients receiving RT/CRT [41–43]. Overall, enhanced knowledge of active and evolving pain mechanisms during RT/CRT is needed for the strategic use of analgesic pharmacology. For example, NSAIDs and mu-opiates may effectively control inflammation and hyperalgesia respectively, more efficiently than gabapentanoids which have a more specific efficiency profile for managing allodynia, a classic manifestation of neuropathic pain [44].

After the use of retrospectively collected clinical data from a large, modern cohort of OC/OPC patients treated with RT, certain limitations apply to our study. These include attrition rates in documented pain descriptors which were not mandatory for reporting. Additionally, analgesic data analysis was restricted to generic groupings of medication prescriptions instead of temporal changes in drug usage. Our next study on algorithmic pain management will incorporate morphine-equivalent drug dosing data to best reflect weekly adjustments in pain medication prescriptions. Analysis of other confounding factors affecting pain experiences such as medical history is warranted. Further prospective data collection to improve the recording of patient medication use data is also needed to improve the accuracy of analgesic use for pain control.

In conclusion, our results demonstrate a significant temporal increase in the severity of pain and other radiation treatment-related acute toxicities throughout the course of RT/CRT in OC/OPC patients and an ongoing need for better and safer pain control in this population. AUC*pain* metrics and the significant impact of different clinical and demographic features on acute pain burden will be used in the future to develop a predictive algorithm for pain management optimization.

## Figures and Tables

**Table 1:** Patients characteristics.

	n	SD/%
**Total**	351	
Age (SD)	58.5	11
Sex (%)		
Males	261	(74%)
Females	90	(26%)
Race (%)		
White or Caucasian	302	(86%)
Black or African American	10	(3%)
Asian	9	(3%)
American Indian or Alaskan Native	3	(1%)
Other/unknown	27	(7%)
Smoking (%)		
Current smoker	21	(6%)
Former smoker	148	(42%)
Never smoker	182	(52%)
Alcohol (%)		
Yes	204	(58%)
No	147	(42%)
Drug abuse (%)		
Yes	64	(18%)
No	287	(82%)
Clinical-T stage (%)		
Tx	3	(1%)
T0	3	(1%)
T1	89	(25%)
T2	129	(37%)
T3	67	(19%)
T4	60	(17%)
Clinical-N stage (%)		
N0	75	(21%)
N1	98	(28%)
N2	170	(49%)
N3	8	(2%)
p16/HPV status (%)		
Negative	38	(11%)
Positive	206	(59%)
Unknown	107	(30%)
Primary tumor type (%)		
Oral cavity	120	(34%)
Oropharynx	228	(65%)
Unknown primary	3	(1%)
Primary tumor site (%)		
Tonsil	112	(32%)
Base of tongue (BOT)	108	(31%)
Tongue	70	(20%)
Buccal mucosa	16	(4%)
Gingiva/GS	12	(3%)
Mandible	7	(2%)
Palate	7	(2%)
Retromolar trigone	7	(2%)
Floor of Mouth	3	(1%)
Alveolar ridge	2	(0.6%)
Lip Gum	2	(0.6%)
Vallecula	1	(0.4%)
Pharyngeal Wall	1	(0.4%)
Unknown	3	(1%)
Treatment (%)		
Concurrent chemoRT	198	(56%)
Induction + concurrent chemoRT	25	(7%)
Induction chemoRT	24	(7%)
RT alone	104	(30%)
Surgery (%)		
Yes	149	(42%)
No	202	(58%)
Survival status (%)		
Alive	318	(88%)
Deceased	43	(12%)

Abbreviations: n: number, SD: Standard Deviation, GS: Gingival Sulcus, chemoRT: chemoradiotherapy, RT: Radiotherapy.

**Table 2:** Patient reported pain trajectory and pain profiles during the weekly see visits (WSVs).

Table:	Baseline	WSV1	WSV2	WSV3	WSV4	WSV5	WSV6	WSV7
Patients reported pain score (n, %)	341 (97%)	351 (100%)	351 (100%)	351 (100%)	330 (94%)	318 (91%)	303 (87%)	159 (45%)
Mean pain score (SD)	1.4 (2.3)	1.1 (1.8)	1.6 (2.0)	3 (2.5)	4 (2.5)	4.2 (2.6)	4.7 (2.7)	5.5 (2.8)
Median pain score	0	0	1	2	4	4	5	5
Pain location (n, %)								
Mouth	33 (10%)	50 (14%)	90 (26%)	181 (52%)	208 (63%)	202 (64%)	200 (66%)	96 (60%)
Throat	27 (8%)	68 (19%)	106 (30%)	167 (48%)	210 (64%)	195 (61%)	255 (84%)	119 (74%)
Skin	0 (0%)	5 (1%)	9 (3%)	38 (11%)	55 (17%)	90 (28%)	105 (34%)	67 (42%)
Other	69 (20%)	39 (11%)	43 (12%)	35 (11%)	34 (10%)	26 (8%)	33 (11%)	16 (10%)
Pain description (n, %)								
Aching	45 (13%)	53 (15%)	53 (15%)	68 (19%)	65 (20%)	64 (20%)	68 (22%)	29 (18%)
Sore	28 (8%)	56 (16%)	81 (23%)	143 (57%)	146 (45%)	156 (49%)	132 (43%)	76 (47%)
Burning	11 (3%)	7 (2%)	11 (3%)	60 (17%)	130 (39%)	140 (44%)	161 (53%)	97 (61%)
Sharp	25 (7%)	6 (2%)	11 (3%)	17 (5%)	24 (7%)	36 (11%)	25 (8%)	21 (13%)
Other	55 (16%)	28 (9%)	35 (10%)	32 (9%)	34 (10%)	31 (10%)	40 (13%)	18 (11%)
Pain onset (n, %)								
Ongoing	45 (13%)	78 (22%)	105 (30%)	158 (45%)	186 (56%)	198 (62%)	203 (67%)	112 (70%)
Gradual	9 (3%)	14 (4%)	13 (4%)	17 (5%)	19 (6%)	10 (3%)	5 (2%)	0 (0%)
Progressive	0 (0%)	0 (0%)	3 (1%)	5 (2%)	9 (3%)	11 (4%)	11 (4%)	9 (6%)
Sudden	4 (1%)	2 (0.5%)	4 (1%)	3 (0.9%)	1 (0.3%)	0 (0%)	2 (0.6%)	0 (0%)
Other	5 (2%)	1 (0.3%)	2 (0.6%)	3 (0.9%)	1 (0.3%)	0 (0%)	1 (0.3%)	1 (0.6%)
Frequency (n, %)								
Intermittent	36 (10%)	69 (20%)	95 (27%)	118 (34%)	136 (41%)	117 (37%)	103 (34%)	52 (33%)
Constant/continuous	56 (16%)	25 (7%)	39 (11%)	74 (21%)	93 (28%)	105 (33%)	126 (42%)	72 (45%)
Progression (n, %)								
Gradually improving	0 (0%)	13 (4%)	10 (3%)	4 (1%)	9 (3%)	8 (3%)	8 (3%)	4 (3%)
Rapidly improving	0 (0%)	0 (0%)	1 (0.3%)	0 (0%)	0 (0%)	1 (0.3%)	0 (0%)	0 (0%)
Gradually worsening	28 (11%)	15 (4%)	38 (11%)	107 (30%)	118 (36%)	132 (42%)	131 (43%)	64 (40%)
Rapidly worsening	0 (0%)	0 (0%)	1 (0.3%)	2 (0.6%)	5 (1.5%)	0 (0%)	4 (1.3%)	1 (0.6%)
Not changed	39 (11%)	57 (16%)	69 (20%)	58 (17%)	64 (19%)	62 (19%)	54 (18%)	44 (27%)

Abbreviations: n = number, % = percentage. Note: the number and the percentages are based on the total number of patients reported pain each week.

**Table 3:** Pain scores according to analgesics subsets.

	WSV1 pain n, median, (IQR)	WSV2 pain n, median, (IQR)	WSV3 pain n, median, (IQR)	WSV4 pain n, median, (IQR)	WSV5, pain n, median, (IQR)	WSV6 pain n, median, (IQR)	WSV7 pain n, median, (IQR)	AUC*pain* scores, median%, (IQR), *p value*
Weak opioids								
No	277, 0, (0–1)	221, 1, (0–2)	148, 2, (0–4)	112, 3, (2–5)	98, 3, (2–6)	80, 4, (2–7)	44, 5, (3–8.75)	9.5%, (4.75–18.25)
Yes	73, 2, (0–4)	127, 1, (0–4)	197, 3, (1–5.5)	217, 4, (2–6)	219, 4, (3–6)	221, 5, (3–7)	115, 5, (4–7)	16.75%, (10.38–23.62)
*P value (Wilcoxon)*	P < 0.001*	P < 0.001*	p < 0.001*	*P* = 0.011*	p = 0.029*	*p* = *0.3158*	*p* = *0.949*	***P < 0.001***
Strong opioids								
No	338, 0, (0–1)	324, 1, (0–3)	303, 2, (1–4)	249, 3, (2–5)	214, 3, (2–5)	190, 4, (2–6)	87, 4, (3–7)	11.5%, (6.50–19.00)
Yes	12, 3, (2–7)	25, 3, (1–4)	46, 4, (1.75–6.25)	80, 6, (4–7.75)	102, 5, (4–8)	113, 6, (4–8)	72, 7, (5–8)	20.5%, (15.00–28.38)
*P value (Wilcoxon)*	P < 0.001*	P < 0.001*	*P* = 0.003*	P = 0.028*	P < 0.001*	p < 0.001*	p < 0.001*	***P*** < ***0.001***
Gabapentin								
No	306, 0, (0–2)	284, 1, (0–3)	274, 2, (1–5)	246, 4, (2–6)	229, 4, (2–6)	220, 5, (2.25–7)	115, 5, (3–7)	15%, (8.5–23.0)
Yes	44, 1, (0–2)	65, 1, (0–3)	76, 3.5, (2–6)	82, 4, (2–7)	87, 5, (2–6)	83, 5, (2–8)	44, 5, (3–8)	17.5%, (11–25)
*P value (Wilcoxon)*	*P = 0.23*	*P = 0.149*	P = 0.007*	*P = 0.657*	*P* = *0.472*	*P = 0.486*	*P* = *0.975*	*P* = *0.062*
Non-opioids								
No	310, 0, (0–1)	299, 1, (0–3)	393, 2, (1–5)	282, 4, (2–6)	272, 4, (2–6)	220, 5, (2.25–7)	146, 5.5, (3–8)	14.75%, (8.5–23)
Yes	40, 2, (0–4)	51, 2, (1–3)	56, 4, (2–5)	48, 4, (2–6	46, 4, (2.75–6.25)	83, 5, (2–8)	13, 5, (3.5–9)	17.5%, (11.5–23.5)
	P < 0.001*	P = 0.005*	P = 0.032*	*P* = *0.808*	*P* = *0.472*	*P = 0.304*	*P* = *0.81*	*P* = *0.07*

Abbreviations: n = number, median = median of pain scores, IQR= Interquartile range of pain scores.
